# Acoustically targeted measurement of transgene expression in the brain

**DOI:** 10.1126/sciadv.adj7686

**Published:** 2024-08-07

**Authors:** Joon Pyung Seo, James S. Trippett, Zhimin Huang, Sangsin Lee, Shirin Nouraein, Ryan Z. Wang, Jerzy O. Szablowski

**Affiliations:** ^1^ Applied Physics Program, Rice University, Houston, TX, USA.; ^2^ Department of Bioengineering, Rice University, Houston, TX, USA.; ^3^ Systems, Synthetic, and Physical Biology Program, Rice University, Houston, TX, USA.; ^4^ Rice Neuroengineering Initiative, Rice University, Houston, TX, USA.

## Abstract

Gene expression is a critical component of brain physiology, but monitoring this expression in the living brain represents a major challenge. Here, we introduce a new paradigm called recovery of markers through insonation (REMIS) for noninvasive measurement of gene expression in the brain with cell type, spatial, and temporal specificity. Our approach relies on engineered protein markers that are produced in neurons but exit into the brain’s interstitium. When ultrasound is applied to targeted brain regions, it opens the blood-brain barrier and releases these markers into the bloodstream. Once in blood, the markers can be readily detected using biochemical techniques. REMIS can noninvasively confirm gene delivery and measure endogenous signaling in specific brain sites through a simple insonation and a subsequent blood test. REMIS is reliable and demonstrated consistent improvement in recovery of markers from the brain into the blood. Overall, this work establishes a noninvasive, spatially specific method of monitoring gene delivery and endogenous signaling in the brain.

## Teaser

Synthetic serum markers can monitor transgene expression in specific brain regions through ultrasound-triggered release.

## INTRODUCTION

Monitoring gene expression in the living brain is crucial for studying its network activity (*1*), diagnosing neurological diseases from onset to progression (*2*), and translating gene therapies into the clinic. For example, formation of memories requires activation of gene expression (*3*). Gene expression is also implicated in pathogenesis of Alzheimer’s disease (*4*). Last, establishing the success of gene therapy delivery is necessary to understand the gene delivery efficacy, durability, and site specificity. However, mapping brain gene expression poses major challenges, especially within the brain.

Typically, measuring gene expression in the brain occurs through tissue-destructive techniques such as biopsy and postmortem histology (*5*). Although common, these approaches are invasive, prohibit repeated measurements, and thus preclude longitudinal assessment of the same subject. Further, they hamper translational and neuroscientific studies, where damage to the brain region being investigated introduces a scientific confound and poses ethical challenges.

In consequence, several noninvasive imaging tools have emerged to detect endogenous gene expression within the brain. These include magnetic resonance imaging (MRI) with genetically encoded contrast agents (*6*–*8*), positron emission tomography (PET) using transgene-binding probes (*9*), optical imaging (*10*–*13*), and biomolecular ultrasound (*14*). These tools are transformative, but also must contend with limitations, including sensitivity constraints, the need for brain-delivered radioactive probes, poor penetration in deep tissues, and signal attenuation through the skull, respectively. Because these techniques rely on penetrant forms of energy to monitor gene expression, they also lack the ability to distinguish large numbers of different signals at once, in contrast to sensitive proteomic (*15*) or transcriptomic (*16*) approaches which can measure millions of molecular species in one sample. Overall, these obstacles have hindered our ability to measure noninvasively and with spatial precision the endogenous gene expression of critical neuronal activity markers, such as immediate early gene *c-Fos*, or expression of multiple genes at once. More sensitive techniques like intravital imaging with luciferases can measure *c-Fos* activity in small animals (*17*), but light generated by luciferases is subject to tissue scattering and skull attenuation which limits their utility in deep brain regions or large animals and prevents accurate localization of the source (*18*).

Recently, focused ultrasound–mediated blood-brain barrier opening (*19*) (FUS-BBBO) was shown to release proteins from the brain (*20*, *21*) into the circulation, in a process called FUS liquid biopsy. This process can measure molecular components within the intact brain tissue, but can only release naturally existing tissue markers. Unfortunately, many gene products are not released into the brain in sufficient quantities to be detectable in the blood following FUS liquid biopsy. To address the issues with limited numbers and sensitivities of natural serum markers, we recently pioneered synthetic serum markers that can report on transgene expression within the brain with a simple blood test. In our method, engineered markers are expressed within the brain, but can cross through the intact blood-brain barrier (BBB) into the bloodstream using the process of reverse transcytosis (*22*). We have shown that these markers, called released markers of activity (RMA) (*23*), originate from the genetically labeled cells in the brain and can inform on transduction or endogenous promoter activity. RMAs are engineered to be highly sensitive—we have shown that as few as ~12 ± 9 cells in the brain need to express RMAs to make them detectable in blood. Because RMAs are proteins, they can be detected in multiplex using any protein chemistry techniques (*24*–*26*). The RMA approach, however, has two important limitations. To achieve spatial precision of monitoring with RMAs, one needs to deliver the genes encoding RMAs to specific brain regions. The simplest way to achieve this is through a direct intracranial injection, which is invasive. Noninvasive gene delivery can be done with BBB-permeable adeno-associated viruses (AAVs) (*27*, *28*) that are injected intravenously, but transduce cells throughout the brain, without spatial precision. Noninvasive gene delivery to specific brain regions can also be achieved with FUS-BBBO but it also requires systemic delivery of AAVs. This results in a second limitation, introduction of a potential for nonbrain tissues to release markers into the blood, confounding the readout.

Here, we present a new paradigm, which resolves both of these limitations and provides a noninvasive method of monitoring gene expression in specific brain regions after noninvasive gene delivery. To achieve this, we combined FUS-liquid biopsy and the concept of RMAs into a new paradigm called recovery of markers through insonation, or REMIS.

Under this approach, designer markers are expressed within the brain in response to gene activity and then released from targeted brain regions into the blood via focused ultrasound application. Our strategy incorporates FUS-BBBO to noninvasively target specific brain regions with millimeter precision, enabling the transport of synthetic markers from the brain into blood. From there, the markers can be detected using conventional biochemical assays such as the enzyme-linked immunosorbent assay (*24*), in vivo detection of luciferases (*18*), and mass spectrometry (*25*, *26*), facilitating routine measurements of gene expression from blood draws.

To establish REMIS, we first expressed markers in neurons under constitutive promoters and confirmed noninvasive transduction at targeted brain regions. The noninvasive transduction was achieved using BBB-permeable AAV, PHP.eB (*27*), that transduces neurons throughout the central nervous system (CNS) after intravenous delivery. Next, we reliably quantified the signals of the markers in the blood following FUS-BBBO. Last, we implemented the markers to measure endogenous neuronal signaling activity. Specifically, we expressed them under the control of a genetic circuit that responds to *c-Fos* when activated by heightened neuronal activity (*29*). We were able to measure the corresponding neuronal activity of the targeted brain regions through blood tests, a process which typically cannot be measured with blood sampling. Overall, our work demonstrates the feasibility of combining genetically encoded reporters and focused ultrasound to noninvasively and specifically measure endogenous gene expression in the brain.

## RESULTS

### Secreted protein reporter as a spatially specific marker for gene expression in the brain

**Fig. 1.**
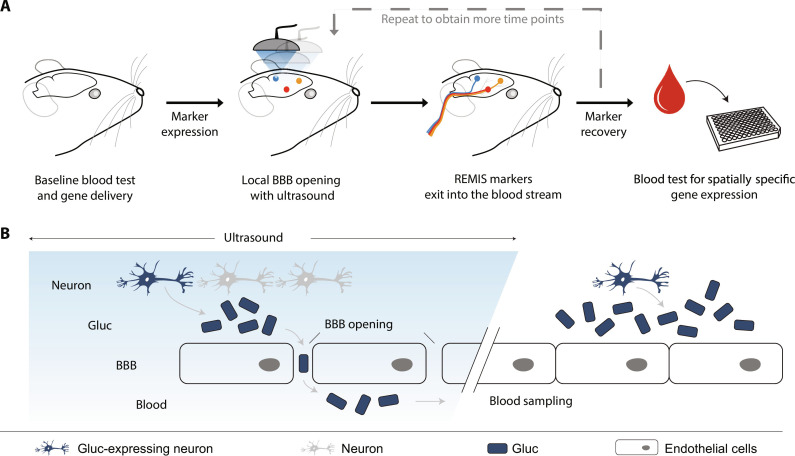
The concept of noninvasive REMIS. (**A**) REMIS uses focused ultrasound to open the blood-brain barrier (FUS-BBBO) and release genetically encoded synthetic markers from the insonated brain regions into the blood. The markers are then collected from simple blood draw for further analysis. Marker levels in the serum can inform on the transgene expression or endogenous physiologic activity in the targeted brain region. (**B**) Any protein that is secreted from the cell can be used as REMIS marker. In the presence of the ultrasound-opened BBB, these secreted markers produced in transduced cells diffuse into the blood. Since ultrasound can be focused with millimeter precision, the markers released into the blood come from a spatially defined brain region. Overall, REMIS process enables noninvasive, spatially specific monitoring of genetically targeted cells in the brain through a simple blood test.

Our REMIS platform relies on engineered protein markers that get expressed in cells and then released into the brain parenchyma. After FUS is used to open the BBB at specific target sites (*30*), the markers are released into the blood, where they can be detected using biochemical methods (Fig. 1). In principle, any serum-detectable protein that can be secreted from cells can be used with REMIS. As our readout, we chose *Gaussia* luciferase (GLuc) for the reporter protein. GLuc is a highly sensitive reporter that emits bioluminescence through an enzymatic reaction. It is also naturally secreted from the cell as it harbors a cell secretion signal peptide (*31*).

**Fig. 2.**
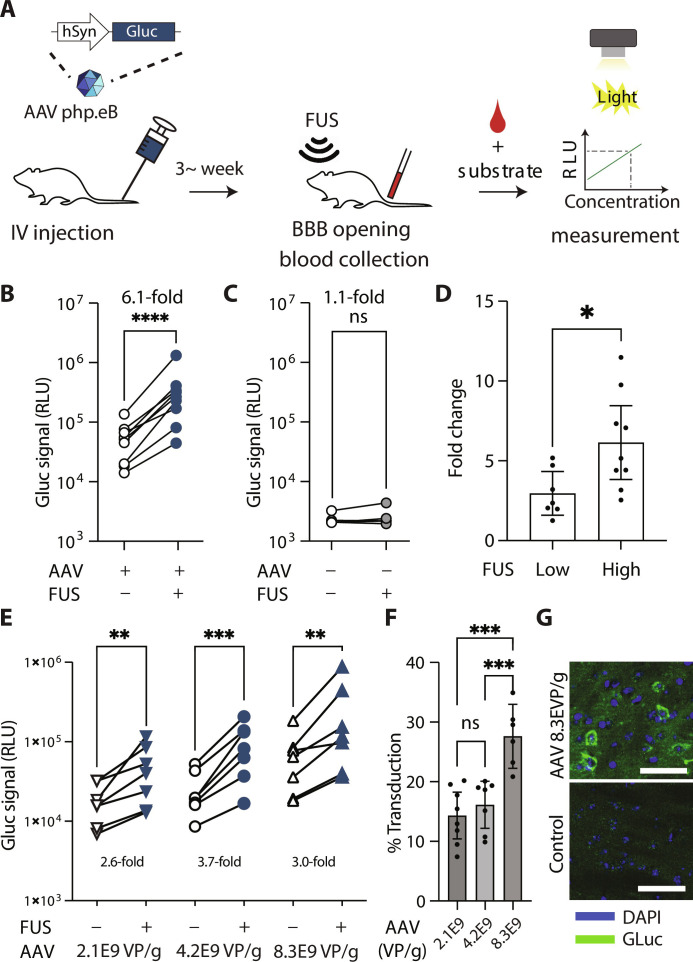
Recovery of GLuc expressed throughout the brain after administration of brain-tropic AAV. (**A**) Schematic for REMIS strategy, from intravenous injection of GLuc-AAV marker to bioluminescence readout of GLuc levels in the blood. (**B**) GLuc bioluminescence signal change before and after FUS-BBBO insonation in mice injected with GLuc-AAV in relative luminescence units (RLU) (*P* < 0.0001; two-tailed, ratio-paired *t* test; *n* = 9). (**C**) Nontransduced mice were used as a control, and show levels of RLU are unaffected by the FUS-BBBO. (*P* = 0.26; two-tailed, ratio paired *t* test, *n* = 5). Serum RLU were measured by integrating bioluminescence signal from serum for 40 s. (**D**) Fold change in GLuc serum luminescence before and after FUS insonation with 0.36-MPa peak negative pressure (PNP) (high) or optimized PNP pressure ranging from 0.27 to 0.33 MPa (low) depending on the targeted areas of the brain (*P* = 0.023; two-tailed, unpaired *t* test). (**E**) FUS-BBBO–induced release of GLuc from the brain for 2.1 × 10^9^ (*P* = 0.0044; ratio-paired *t* test; *n* = 7), 4.2 × 10^9^ (*P* = 0.0002; ratio-paired *t* test; *n* = 7), and 8.3 × 10^9^ (*P* = 0.0023; ratio-paired *t* test; *n* = 7) viral particles per g of body weight using optimized peak negative pressures (ranging from 0.27 MPa to 0.33 MPa). (**F**) Comparison of transduction efficiency as measured by brain histology at the three AAV doses (*P* < 0.001, one-way ANOVA; *n* = 8, 7, 6, for low, medium, and high AAV dose groups, respectively; Tukey post hoc test was used for pairwise comparisons; high dose versus medium, *P* = 0.0009; high dose versus low: *P* = 0.0002; medium dose versus low: *P* = 0.743). (**G**) Representative sections showing subcellular distribution of GLuc immunostaining (green) and nuclear stain 4′,6-diamidino-2-phenylindole (DAPI; blue). Scale bars, 50 μm. ns > 0.05, *P < 0.05, **P < 0.01, ***P < 0.001, and ****P < 0.0001.

To monitor the neuronal activity in specific brain regions, we constructed an AAV vector expressing GLuc under the control of the Synapsin 1 (hSyn1) promoter, which drives specific transgene expression in neurons. As an initial proof of concept, we delivered the hSyn1-GLuc vector to the entire mouse brain using a BBB-permeable, brain-specific AAV serotype called PHP.eB (*32*, *33*) (GLuc-AAV) and evaluated subsequent GLuc levels in the blood after FUS-BBBO insonation (Fig. 2A). Here, we intravenously injected mice with GLuc-AAV at 8.3 × 10^9^ viral particles per gram of body weight following the dose from our previous studies (*30*, *34*). Afterward, we measured GLuc bioluminescence levels while the BBB was still intact to obtain baseline GLuc signal levels. Next, we insonated mice with FUS-BBBO using a high peak negative pressure of 0.36 MPa against eight specific brain regions within the striatum, thalamus, midbrain, and ventral hippocampus, which was within the range of pressures used for BBB opening in our previous studies (*30*, *34*–*36*). Last, we collected a blood sample after 7.5 min, which is the shortest time feasible for blood collection.

Our results showed that all GLuc-AAV^+^ mice had increased GLuc signals in the plasma after insonation, with a mean fold change of 6.1 ± 2.1 [95% confidence interval (CI); *P* < 0.0001, ratio-paired *t* test; *n* = 9] (Fig. 2B). In the nontransduced control, we observed no changes in GLuc serum levels (*P* = 0.26, ratio-paired *t* test; *n* = 5).

To confirm BBB opening after insonation, we performed Evans blue dye (EBD) extravasation in the GLuc-AAV^+^ mice. On average, 7.3 ± 0.4 of eight targeted brain sites in each mouse showed successful EBD delivery (95% CI; 92% of successful opening 66 of 72 of the targeted sites, *n* = 9) (fig. S1). These regions represented approximately 4% of the brain volume in total. Together, our results thus far suggest that GLuc is successfully transported from the brain into the blood after insonation.

Next, although FUS-induced tissue damage typically self-resolves within hours to days (*37*) and results in no neuronal loss (*35*), we sought to minimize the potential for tissue damage while maintaining successful BBB opening and marker release. Such damage typically presents with a extravasation of small numbers red blood cells (RBCs) (*19*, *38*, *39*) at lower pressures, which can progress to gross damage that is visible in histology, such as vacuolation, or visible hemorrhage when higher pressures are used (*40*, *41*). Thus, we focused on the damage defined as the presence of submillimeter-scale pockets of RBCs extravasation that typically observed after FUS-BBBO (*39*). We graded this damage based on the size of the bounding ellipse within which all of the damage can be found and defined five classes of damage: no damage (0 μm), damages below 100 μm, between 100 and 200 μm, and then either between 200 and 400 μm or greater than 400 μm. Typically, only a fraction of areas within that bounding box showed any discernible damage, both in our current study (fig. S2B) and previously published work (*39*). We chose the peak negative pressure levels to be 0.33 MPa in the striatum, 0.30 MPa in the thalamus and midbrain, and 0.27 MPa in the ventral hippocampus to enable successful opening in at least seven of eight targeted sites in each mouse, while minimizing the presence of damage in the highest damage category (fig. S2C). We decided to use the lowest tested pressure for thalamus, where damage was present regardless of the tested pressure. Unless otherwise noted, we used these pressure levels for subsequent experiments.

We then investigated marker release before and after insonation under the optimized pressure levels and compared the marker release with 0.36 MPa (high) for each brain region. We found that lower optimized pressures still released GLuc, albeit at a lower fold change over the baseline compared to high pressure (3.0 versus 6.1 arithmetic mean fold change for low and high pressure, *P* = 0.0023, unpaired two-tailed *t* test; Fig. 2D). However, lower pressure still resulted in highly significant release of the REMIS markers from the brain (Fig. 2e, left-most panel, *P* = 0.0002, *n* = 7, ratio-paired *t* test).

Afterward, to evaluate whether transgene transduction efficiency affects GLuc signal levels in the serum post-insonation, we injected mice with GLuc-AAV at different doses (8.3 × 10^9^, 4.2 × 10^9^, and 2.1 × 10^9^ viral particles per gram of body weight). After 3 to 4 weeks of expression, we tested GLuc serum levels before and after insonation. We found that, for each dosage group, all mice exhibited enhanced GLuc serum levels after insonation (Fig. 2E). To measure the significance of the ratio of change before and after insonation, we used a ratio-paired *t* test. The fold changes compared to baseline were significant for each experiment injected with low-, medium-, or high-dose AAV: 2.6-fold ±1.3 (*P* = 0.0044, *n* = 7), 3.7-fold ±1.2 (*P* = 0.0002, *n* = 7), and 3.0-fold ±1.1 (*P* = 0.0023; *n* = 7) (mean, 95% CI; ratio-paired *t* test), respectively. Moreover, the ratio of post-insonation GLuc levels to baseline levels was unaffected by viral dose [fig. S3, *P* = 0.4184; *F* = 0.9148; one-way analysis of variance (ANOVA)]. Further, histologic measurements of brain sections showed that high-dose AAV mice resulted in 27.6% GLuc-positive cells, which was significantly higher than values observed with either low and medium viral doses (14.3 and 16.1%, *P* = 0.0009 and *P* = 0.0002, respectively) (*P* = 0.0001; *F* = 15.45; one-way ANOVA, pairwise comparisons P < 0.001 when comparing high dose to lower doses, or P > 0.05 otherwise; Fig. 2F). These results suggest, that ultrasound pressure, rather than the transduction efficiency, determines the efficiency of transgene product release from the brain. The absolute levels of post–FUS-BBBO luminescence normalized to mouse weight showed positive correlation of *R* = 0.55 with the transduction levels (fig. S4). Representative images showing transduction of GLuc are shown in Fig. 2G.

**Fig. 3.**
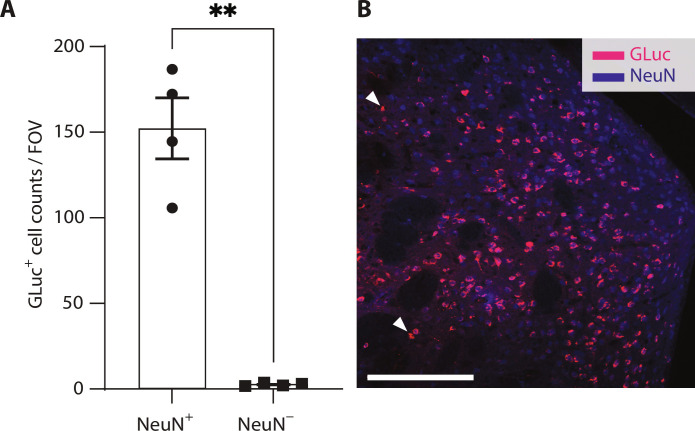
Cell-type specificity of REMIS. (**A**) We coimmunostained GLuc and the neuronal marker (NeuN) to quantify the number of cells per the field of view (FOV) expressing GLuc that are neurons (98.2% ± 1.1%, 95% CI, paired *t* test, *P* = 0.0037). (**B**) Representative image of the brain section showing GLuc (magenta) and NeuN immunostaining (blue). Two of the cells positive in GLuc (GLuc^+^) and negative in NeuN (NeuN^−^) are designated with an arrowhead. Scale bar, 200 μm. ns > 0.05, *P < 0.05, **P < 0.01, ***P < 0.001, and ****P < 0.0001.

Last, to confirm cell type specificity of *hSyn1* promoter we performed coimmunostaining for the expression of GLuc marker and the marker for neurons found within the targeted areas (NeuN) (*42*). We found that 98.2% ±1.1 (95% CI) of GLuc-transduced cells were neurons (Fig. 3A). Representative images along with the examples of cells counted as NeuN^−^ are shown in Fig. 3B with arrowheads.

### Released marker recovery pharmacokinetics after insonation

**Fig. 4.**
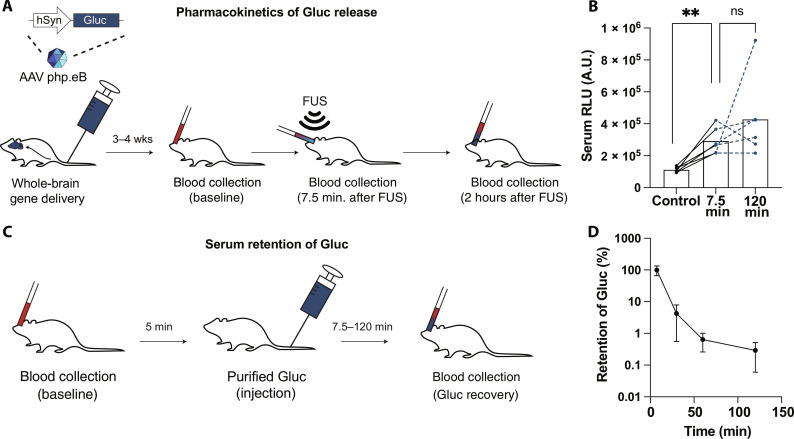
Pharmacokinetics of marker release in REMIS. (**A** and **B**) GLuc bioluminescence level measured at pre-insonation baseline, 7.5 and 120 min after FUS-BBBO insonation. The resultant levels of GLuc are influence by both the release of GLuc from the brain, and its concurrent clearance from the serum. (ns; ratio-paired *t* test; *n* = 5). (**C** and **D**) To measure the clearance rate from the serum, purified GLuc protein was administered intravenously to measure in vivo serum half-life of the protein in the blood. Blood was collected before injection as the baseline and then again at 7.5, 30, 60, and 120 min after injection to calculate the half-life (*n* = 30 blood collections across 15 mice). ns > 0.05, *P < 0.05, **P < 0.01, ***P < 0.001, ****P < 0.0001.

To evaluate the pharmacokinetics of marker released from the brain, we collected blood samples before and after insonation from GLuc-AAV^+^ mice (Fig. 4A). GLuc signal levels showed no statistically significant difference between 7.5 and 120 min after insonation (Fig. 4B). This result could be explained by several possibilities, including the persistent release of markers from the brain and a marker half-life in the blood that is long compared to the duration of marker release from the brain.

To explore the possibility of a long marker half-life in the blood, we intravenously injected purified GLuc protein into mice and collected blood at different time points from 7.5 to 120 min (Fig. 4C). We found that the GLuc half-life from a single exponential decay was 7.6 ± 2.4 min (SD; *n* = 30 blood collections in *n* = 15 mice) (Fig. 4D), with over 99.4% of the GLuc eliminated by 60 min after injection. Rather than a long half-life, this result suggests a continuous release of GLuc from the brain over time, with serum levels of GLuc replenishing over 120 min, enabling a broad time window for blood collection for this marker.

Recognizing that GLuc had a short half-life in serum, we proceed to evaluate whether the release of markers stops over longer periods of time. We chose a time point of 48 hours, which is after a typical BBB closure time for FUS-BBBO procedures [6 to 24 hours; (*43*, *44*)]. As before, we performed a baseline blood collection from GLuc-AAV^+^ mice. Next, we performed FUS-BBBO and collected the second blood sample within 7.5 min. Since we aimed to collect the blood 48 hours after the insonation, we were unable to confirm the BBB opening histologically through the EBD extravasation as in our other experiments. However, of *n* = 6 tested mice, *n* = 5 showed an increase in the serum GLuc following insonation, suggesting a successful FUS-BBBO. This success rate is consistent with our previous studies (*30*) and other experimental groups in this study. Considering that the main goal of the experiment was to evaluate whether the increased signal could return to baseline over time, we proceeded with the analysis of the mice that showed increased GLuc signal after the BBB opening. Among these five mice, we observed 1.9 ± 0.5-fold (mean with 95% CI, *P* = 0.0068; two-tailed, ratio-paired *t* test) increase in the GLuc levels in the serum following insonation, which was significantly higher than the baseline. After 48 hours, we collected the final blood sample and found a significant reduction in the serum marker levels compared to the post–FUS-BBBO peak (2.4 ± 0.3-fold, mean with 95% CI, *P* < 0.0001; two-tailed, ratio-paired *t* test). The GLuc levels at 48 hours were comparable to pre-insonation baseline (0.8 ± 0.2-fold, mean with 95% CI, *P* = 0.14; two-tailed, ratio-paired *t* test). The absolute levels of GLuc in the serum can be calculated using the standard curve in fig. S6.

### Noninvasive measurement of neuronal activity in specific brain regions

**Fig. 5.**
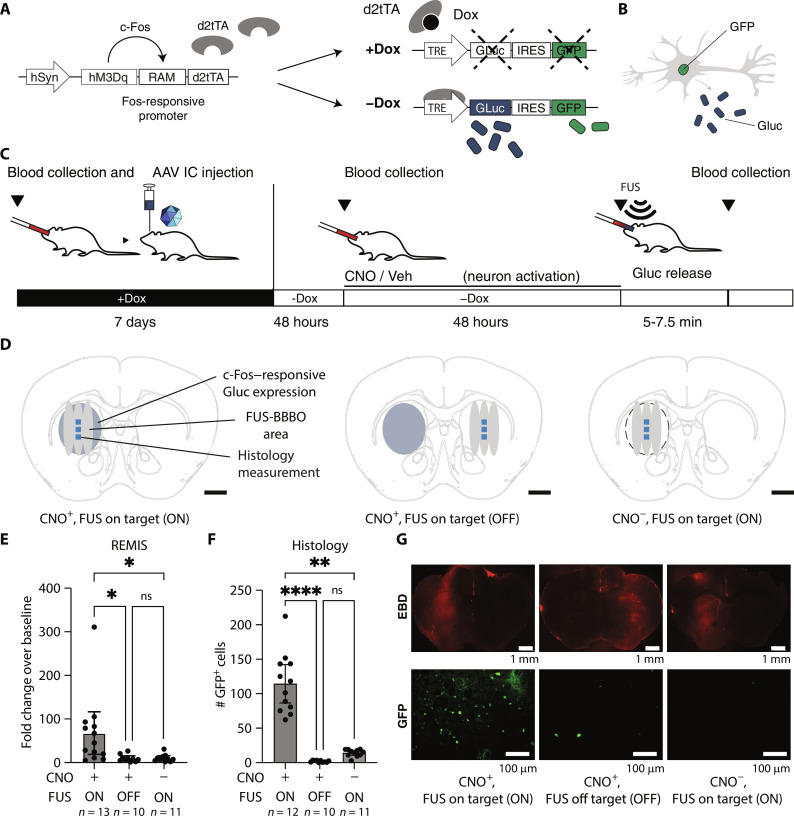
Noninvasive measurement of c-Fos in specific brain regions with REMIS. (**A**) We used rapid activity marking (RAM) system to drive GLuc expression after prolonged neuronal activity. To ensure temporal precision of c-Fos recording, we shut down the RAM system with Dox until the beginning of recording. As an independent control for measuring c-Fos, we also expressed GFP under internal ribosome entry sites (IRESs). We drove neuronal activity with hM3Dq DREADD. (**B**) Upon activation, neurons express and release GLuc into the brain interstitium but express GFP intracellularly. (**C**) We drew blood as a baseline and injected AAVs carrying GLuc expressed under RAM system into unilateral caudate putamen in mice. We provided Dox in food for 7 days to express RAM system components, while preventing *c-Fos*–responsive production of GLuc. We then withdrew Dox, collected another blood sample after 48 hours, and administered CNO or a vehicle control intraperitoneally. After 48 hours, we performed FUS-BBBO to release GLuc into the blood. (**D**) Illustration of on- and off-target FUS-BBBO sites in the striatum for each group (gray), activated *c-Fos* (blue oval), and sites of histological measurements (blue squares). (**E**) GLuc levels in the serum after opening the BBB of target regions (*P* = 0.0032; Kruskal-Wallis test; CNO^+^ FUS on target versus CNO^+^ FUS off target: *P* = 0.0104; CNO^−^ on target versus vehicle, *P* = 0.0137; CNO^+^ FUS off target versus vehicle: *P* > 0.9999). (**F**) Histological measurement of GFP expressed under IRES in response to c-Fos accumulation. (*P* = 0.0001; Kruskal-Wallis test; CNO^+^ FUS on target versus CNO^+^ FUS off target: *P* < 0.0001; CNO^−^ on target versus vehicle, *P* = 0.0099; CNO^+^ FUS off target versus vehicle: *P* = 0.0636). (**G**) Representative images showing the EBD signal (top) or the GFP expression in the tissue sections (bottom). ns > 0.05, *P < 0.05, **P < 0.01, ***P < 0.001, and ****P < 0.0001.

We next sought to determine whether REMIS could be used to measure endogenous neuronal activity in the brain. Here, we constructed a conditional genetic circuit to tether GLuc expression to neuronal activity (Fig. 5A). To gain temporal control over GLuc, we used designer receptor exclusively activated by designer drug (DREADD) hM3Dq (*45*), which induces neuronal firing when turned on by the DREADD agonist clozapine-*N*-oxide (CNO) back-metabolized to clozapine at subthreshold doses (*9*). We chose CNO as the DREADD ligand to take advantage of multihour timeline of drug action following the single administration compared to clozapine (*46*). We also incorporated into the circuit the doxycycline (Dox)–dependent Tet-Off system called rapid activity marking (RAM) (*29*), which requires both an active *c-Fos*–responsive promoter and the absence of Dox to drive GLuc expression that is then released into the interstitial space of the brain and a nuclearly localized green fluorescent protein (GFP) under internal ribosome entry site (IRES) as a histological ground-truth control of *c-Fos* activation (Fig. 5B).

To activate neurons, we injected AAVs carrying the entire *Fos*-responsive RAM-controlled (*29*) GLuc expression system into the left caudate putamen of mice. We then administered Dox (P.O. in food ad libitum) for 7 days to suppress GLuc expression until a specified window needed for neuronal activity recording. Then, 48 hours after withdrawing Dox, we collected a baseline blood sample (Fig. 5C). Immediately following, we injected CNO at a previously validated dose (*45*) intraperitoneally to drive neuronal activation and the coupled expression of GLuc. CNO, a prodrug (*9*, *46*) for the DREADD activator in vivo, was chosen over other chemogenetic activators due to its long timeline action (*45*), well-suited to long-term recording with the RAM system (*29*). As a control, we also injected a group of mice with vehicle to evaluate baseline levels of GLuc signal in the absence of CNO-induced neuronal activity. After 48 hours (*29*, *47*), we insonated mice at a target site within the striatum (*n* = 10 for vehicle and *n* = 13 for CNO^+^ mice) (Fig. 5D, left and middle). To test the site specificity of REMIS, we also insonated a group of CNO^+^ mice in the contralateral striatum (*n* = 11) (Fig. 5D, right).

Following on-target insonation, we found that DREADD-activated neurons in CNO^+^ mice showed 67.6-fold ±44 (mean with 95% CI) increase of GLuc in the blood. This enrichment was higher than that observed in both the vehicle group (8.3-fold ±3.1, mean with 95% CI, *P* = 0.0137, Kruskal-Wallis test, with multiple comparisons) and off-target CNO^+^ group (8.1-fold ±6.3, mean with 95% CI, *P* = 0.0104, Kruskal-Wallis test, with multiple comparisons) (Fig. 5E). Moreover, targeting the contralateral site was not significantly different from targeting the striatum in the vehicle control (*P* > 0.9999, Kruskal-Wallis test), showing site specificity of REMIS.

We then examined whether measurements of neuronal activation using REMIS (Fig. 5E) could be recapitulated histologically. The *c-Fos*–responsive promoter (*29*) of our genetic circuit is designed to drive concurrent expression of secreted GLuc and intracellular GFP in response to induced neuronal activity (Fig. 5, A and B). Our data showed that CNO^+^ mice that expressed GLuc in the striatum had the highest number of GFP-positive cells within the insonated target areas when compared to the off-target mice and vehicle controls (Fig. 5F). Specifically, we found an 8.0-fold difference in the number of GFP-positive cells between on-target CNO^+^ mice and the vehicle control (*P* < 0.0001, Kruskal-Wallis test), which corresponds to the 8.1-fold difference between these two groups when GLuc was measured using REMIS (Fig. 5E). Representative images highlighting the area of BBB opening and GFP expression for each group is shown in Fig. 5 and additional BBB opening examples in fig. S7. These results suggest that REMIS can measure *c-Fos* activation in specific brain regions.

## DISCUSSION

Together, our results establish REMIS as a paradigm for noninvasive, site-specific measurement of transgene expression in genetically targeted cells. Instead of directly measuring gene expression within the deep tissue, which is difficult to achieve, REMIS provides a means of recording reporter concentration in an ultrasound-defined region by transporting engineered markers of gene expression into the blood where they can be easily quantified. Our results demonstrate suitability of this approach for confirmation of gene delivery and investigating cellular physiology, specifically for monitoring sustained neuronal activation levels that give rise to *c-Fos* activity.

Compared to existing approaches, REMIS has several advantages. Traditionally, brain biopsy or postmortem histology is used to extract cells from the brain and measure gene expression. However, they are invasive and destructive to the tissue. In consequence, they cannot record gene expression activity over multiple time points and they damage the very brain region being studied. REMIS represents a nondestructive and potentially repeatable (*48*) alternative to biopsy-based measurement of gene expression.

Second, whereas imaging methods such as MRI with genetically encoded contrast agents can visualize the entire brain, REMIS enables monitoring of a predefined brain region with millimeter precision. REMIS, however, has two important advantages. First, it achieves over an order of magnitude higher signal to baseline ratios than observed for genetically encoded MRI contrast agents (*6*, *7*, *49*, *50*). In addition, REMIS measures molecule concentration through biochemical blood testing, enabling inexpensive detection of multiple types of molecules, possibly in a single sample.

Compared to PET, REMIS does not rely on the development of transgene-binding radioactive BBB-permeable probes, while maintaining comparable spatial precision. When compared to optical imaging, REMIS allows measurement of markers in any brain region that is accessible with focused ultrasound, including behind thick skulls in various species or in deep brain regions (*30*, *48*, *51*, *52*) both locally or brain-wide (*35*, *53*). In contrast, for optical methods, such as fluorescence or bioluminescence imaging, depth of penetration and scattering of light through the skull or tissue are major technical obstacles. Thus, REMIS has both an advantage over optical methods and future utility in large-animal species and deep or large brain regions.

Compared to RMAs (*23*), REMIS appears less sensitive with lower fold changes over the baseline and requires ultrasound procedure before readout. In contrast, RMAs cross through an intact BBB on their own, allowing for a simpler readout. However, RMAs rely on the site-specific gene delivery to determine the spatial precision and average signal over the entire transduced cell population. REMIS, on the other hand, can sample different brain sites within the transduced cell populations, providing it with unique advantages. For example, REMIS can be used for validating gene delivery sites, assessing a diffusion of secreted transgenes (*54*, *55*), or monitoring gene expression in individual brain sites. Current brain gene therapies struggle with monitoring gene expression without invasive procedures. REMIS offers a nonsurgical, nontissue-destructive solution by augmenting gene therapy to express detectable REMIS markers, akin to using fluorescent proteins in histology. REMIS also could enable long-term spatially specific transgene expression monitoring, without causing tissue damage, as shown by safety of repeated applications of FUS-BBBO in large animals (*48*). Our results showing noninvasive monitoring of *c-Fos* in a specific brain region could also be applied to a range of studies. Immediate early genes such as *c-Fos* and *Arc* are activated by heightened neuronal activity (*56*) and, thus, REMIS could be used to point to a brain region of interest and measure long-term changes in neuronal activity, for example, to determine successful neuromodulation, or activation neuronal ensembles associated with learning (*57*) and memories (*58*).

The spatial resolution of REMIS is dependent on the parameters of focused ultrasound for the BBB opening and ranges from millimeter to submillimeter precision (*30*, *52*). In humans, current devices open the BBB with millimeter precision (*52*). One possibility that could reduce the resolution is diffusion of the markers away from the site of their expression. To ensure that the expression is highly localized, we opted for a direct intraparenchymal AAV delivery to express GLuc in unilateral striatum. Our experiments suggest that the spatial diffusion of markers is limited in the tested scenario. We measured *c-Fos* activation in the striatum contralateral to the area of activation (distance of ~2.5 mm) and found no diffusion of markers into that region. Moreover, the application of FUS-BBBO to the striatum contralateral to the CNO-activated brain region had an effect that was indistinguishable from opening the BBB in vehicle control mice (Fig. 5E). These data suggest that extravasation of REMIS markers from the brain is localized within distance on the order of about millimeters. Our data also show that REMIS can be applied after intraparenchymal delivery, which is commonly used to achieve spatially directed AAV delivery in various species (*59*). Future studies will require further investigation into the spatial precision of REMIS. For example, the diffusivity of a particular reporter can affect the spatial precision if the proteins diffuse from neighboring brain regions into the FUS-BBBO site. In addition, the exact time at which the blood is collected after FUS-BBBO may also affect the spatial precision. Over time, proteins from farther away can reach the FUS-BBBO site and escape into the circulation.

The spatial resolution of REMIS could also contribute to variability when measuring absolute levels of brain transduction between individual animals. For example, white matter previously has shown lower level of FUS-BBBO efficacy than the gray matter (*60*). Thus, where FUS-BBBO is targeted and how much of the white matter that site contains could affect the absolute levels of REMIS markers released into the serum. To avoid this variability, tracking the gene expression over time within the same site would normalize site-specific effects of REMIS recovery.

REMIS’s cell type specificity is reliant on the genetic targeting. In our results, we used synapsin promoter that restricts expression of the REMIS markers to neurons and found that their expression was highly specific (Fig. 3). The fold change of REMIS markers in the serum is dependent on the ultrasound pressure (Fig. 2D), rather than the viral dose (fig. S3). The process of marker recovery was robust; of note, every single mouse tested in this study has shown an increase in the REMIS marker levels after the BBB opening, reaching high levels of significance even for lower fold changes (Fig. 2E). These results suggest that REMIS could be successfully used in small cohorts of animals, which is critical for large animal studies, or any potential clinical applications.

Last, the temporal resolution of REMIS is largely dependent on the temporal profile of gene expression driving it. The release of markers from the brain itself is rapid with markers appearing in serum within minutes (Fig. 4, A and B). The c-Fos (*45*) expression, on the other hand, occurs over minutes to hours timescale, with RAM system needing 24 to 48 hours for detectable expression (*29*). For the convenience of measurement, we saw a steady, continuous, release of the GLuc over at least 120 min. Steady release makes the experimentation less time dependent and thus more robust. Other markers could be designed that exit the brain more rapidly depending, for example, on their size and diffusivity or binding affinity within the extracellular matrix of the brain.

An interesting aspect is the presence of baseline GLuc in the serum even in the absence of BBB opening. One possibility is that GLuc is cleared through the glymphatic system and eventually enters the blood (*61*). It is also possible that the PHP.eB, while specific to the CNS, may exhibit low levels of peripheral neuron transduction that is sufficient to produce baseline GLuc levels (*62*) observed. The third possibility is the passage of GLuc through the BBB, for example, through nonspecific interaction of GLuc with FcRN (*63*) or being encapsulated within the transcytosing endosomes by proximity. Regardless of the mechanism, REMIS reading is independent of the baseline, as it observes changes in serum marker levels over the baseline in response to opening the BBB in a specific brain site. This feature is critical in cases when one cannot confirm the origin of the markers in the serum.

The REMIS system could be made more powerful with improvements to each of its components: FUS-BBBO, methods of gene delivery to the brain, gene circuits measuring endogenous gene expression activity, or methods of serum marker detection. For example, real-time monitoring of cavitation (*64*–*67*) has been widely used in human FUS-BBBO (*37*) studies to optimize the ultrasound pressures for safety and account for differences in skull attenuation. Similar recovery of markers could also be used for others parts of the CNS such as the eye (*68*) or spinal cord (*69*). Design of new AAV vectors can lead to improved noninvasive delivery. For example, AAVs can be designed to pass through an intact BBB after a systemic injection and specifically transduce the CNS (*28*) or be more efficiently and tissue-specifically delivered with FUS-BBBO (*34*). They can also be optimized to work in different species (*70*, *71*). These noninvasive gene delivery tools synergize with REMIS, which enables similarly noninvasive measurement of the success of gene delivery. Last, design of new gene circuits (*29*, *72*, *73*), molecular recorders (*74*, *75*), or mRNA sensors (*76*, *77*) that can translate cellular physiology into gene expression will enable new applications of noninvasive techniques like REMIS. REMIS uses biochemical testing, which opens up the possibility of measuring multiple types of markers simultaneously, potentially enabling multiplexed monitoring through detection techniques such as mass spectrometry proteomics (*78*) or single-molecule protein sequencing (*79*, *80*). Such multiplexed imaging is not currently possible with MRI, PET, or even with optical methods, where the number of colors in in vivo imaging is orders of magnitude fewer than what can be detected through serum sampling with proteomic techniques.

These improvements will facilitate the development and translation of REMIS as a paradigm for precise noninvasive monitoring of genetically targeted cell populations within specific brain regions.

## METHODS

### Animals

Wild-type C57BL/6J (strain no. 000664) male and female mice were obtained from Jackson Laboratory (Bar Harbor, ME). Animals were housed in a 12-hour light-dark cycle and were provided with food and water ad libitum. Mice were selected randomly for experiments, ensuring that each cage was represented in multiple comparison groups to avoid cage-specific effects. All mice for which it was possible to obtain sufficient amount of blood or tissues were included in the analysis. All experiments were conducted under a protocol (#IACUC-23-138-RU) approved by the Institutional Animal Care and Use Committee of the Rice University and ARRIVE guidelines were followed during the reporting.

### Plasmid construction

To construct AAV-*hSyn-GLuc*, the vector AAV-*hSyn*-*GLucM23-iChloC-EYEP* (Addgene #114102) was digested with Spe I and Eco RV (New England Biolabs) to isolate the backbone containing the hSyn promoter. GLucM23, a GLuc variant, was amplified by polymerase chain reaction from the same vector and its DNA was extracted using the Monarch DNA Gel Extraction Kit (New England Biolabs, Ipswich, MA). GLuc was inserted into the digested backbone through Gibson Assembly Hi-Fi kit (New England Biolabs, Ipswich, MA). AAV-*hSyn-hM3Dq*-RAM-*d2tTA* was constructed as previously described (*23*). To construct AAV-RAM-*GLuc-IRES-GFP*, the GLuc DNA was amplified from the AAV-TRE-RMA-IRES-GFP plasmid in our previous work and assembled into the same backbone after digestion using Pme I and Bam HI (*23*). AAV-*hSyn*-*CLuc *was constructed similarly by amplifying the CLuc DNA from CLuc-RMA (CLuc-Fc) plasmid and inserted into the same backbone after digestion using Eco RV and Bam HI.

### AAV production

PHP.eB (*27*) AAV was packaged with the AAV-*hSyn-GLuc* plasmid construct using a commercial service (Vector Builder, Chicago, IL) and the titer was provided by the manufacturer.

### Intravenous administration of AAVs

AAV was injected intravenously. Mice at 10- to14-week-old were anesthetized with 2% isoflurane in oxygen and then cannulated in the tail vein using a 30-gauge needle connected to PE10 tubing. The cannula was then flushed with heparin (10 U ml^−1^; Sigma-Aldrich) in sterile saline (Hospira) and affixed to the mouse tail using a tissue adhesive. Subsequently, the mice were injected via tail vein with PHP.eB AAV (2.1 to 8.3 × 10^9^ viral particles per gram of body weight) encoding GLuc under the *hSyn* promoter. AAV-injected mice were housed for 3 to 4 weeks to allow for gene expression.

### Focused ultrasound BBB opening

Mice at 14- to 18-week-old were anesthetized with 1 to 2% isoflurane in oxygen. The hair on mice head was removed by shaving with a trimmer. The mice were then cannulated in the tail. Subsequently, the mice were placed on a stereotaxic mount, with their heads held in place with a custom-made plastic nosecone and secured with ear bars. Bregma and Lambda markers were used to target ultrasound to specific brain regions using a stereotaxic frame (RK-50, FUS Instruments). Ultrasonic transducer was coupled to the shaved area of the head via degassed water in water bath and degassed aquasonic ultrasound gel. The mice were injected with approximately 2.8 × 10^6^ DEFINITY microbubbles (Lantheus) dissolved in sterile saline, per gram of body weight for each targeted site. For each targeted site, DEFINITY was reinjected before insonation. The ultrasonic parameters used were 1.5 MHz, 10-ms pulse train length, and 1-Hz pulse repetition frequency for 120 pulses. The pressure for insonation was varied between experiments and targeted sites based on evaluation of safety and efficacy of BBB opening in this study. The range of peak negative pressure used in this study was 0.27 to 0.36 MPa, as calibrated by the manufacturer (FUS Instruments, Toronto, Canada). In the GLuc recovery experiment, targeted sites represented 4% of the brain volume in total, as measured by full-width half-maximum pressure of the FUS field. Because of equipment failure, experiments on c-Fos measurement used a new transducer with a different calibration curve. BBB opening and tissue damage were used to confirm the validity of new calibration in vivo and used manufacturer-calibrated pressure of 0.46 MPa, which is not directly comparable with the pressures used in previous experiments due to differences in manufacturer’s (FUS instruments, Toronto, Canada) calibration methods. However, the opening was present and comparable to previous experiments (Fig. 5G) with 91.2% (31 of 34) mice showing opening (fig. S7) and we observed no apparent damage in *n* = 12 randomly selected mice (*n* = 4 per group for CNO with on target FUS-BBBO, CNO with off target FUS-BBBO, and vehicle with on-target FUS-BBBO). After ultrasound insonation, aquasonic ultrasound gel was removed and the incision was closed. EBD (5%) dissolved in 1× phosphate-buffered saline (PBS) was injected intravenously. EBD passes selectively through a permeable BBB and localizes in the brain parenchyma, allowing visualization of where the BBB has been opened (*81*). Successful BBB opening showing EBD extravasation was a criterion for inclusion in the FUS-BBBO groups, except for mice in fig. S5, where histology to assess BBB opening was not possible due to terminal blood collection occurring after the expected BBB closure. After 20 min, mice were perfused with 10% neutral-buffered formalin (Sigma-Aldrich) after displacing blood with 1× PBS (10 U ml^−1^) via cardiac perfusion.

### Pharmacokinetic analysis of GLuc serum half-life

Purified GLuc protein (Nanolight Technology) was injected intravenously through a tail vein catheter. C57Bl6j mice at 10- to 14-week-old were injected with purified GLuc protein in 1× tris-buffered saline under isoflurane anesthesia (1 to 5% in oxygen). After a period of 7.5 to 120 min, mice were again anesthetized in 2% isoflurane in oxygen. Afterward, one to two drops of 0.5% ophthalmic proparacaine were applied topically to the cornea of an eye and a heparin-coated microhematocrit capillary tube (Thermo Fisher Scientific) was placed into the medial canthus of the eye and the retro-orbital plexus was punctured to withdraw 50 μl of blood. Each mouse underwent a maximum of three blood collections, baseline measurement and measurement at 7.5 min followed by one additional collection of which the last was terminal. The collected blood was centrifuged at 5000*g* for 15 min to isolate plasma and stored at −20°C until use.

#### Luciferase assay

To conduct luciferase assay, 15 μl of plasma was placed in a black 96-well plate. Bioluminescence of GLuc was measured by injecting plasma samples with 50 μl of 20 μM coelenterazine (Nanolight Technology) dissolved in luciferase assay buffer using an injector in a Tecan M200 microplate reader (Männedorf, Switzerland). The luciferase assay was calibrated to the standard curve with purified GLuc protein (Nanolight Technology), using the same protocols as in the pharmacokinetics analysis, in mouse serum (GeneTex).

### Immunostaining

Mice brains were extracted and postfixed in 10% neutral-buffered formalin overnight at 4°C. Coronal sections were cut at a thickness of 50 μm using a vibratome (Leica) and stored at 4°C in 1× PBS. Sections were stained as follows: (i) preincubate with permeabilizing agent (1% Triton X-100 and 0.5% Tween 20 in 1× PBS) for 1 hour; (ii) block for 1 hour at room temperature with blocking buffer (0.3% Triton X-100 and 10% normal donkey serum in 1× PBS); (iii) incubate with primary antibody overnight at 4°C; (iv) wash in 1× PBS for 10 min three times; and (v) incubate with secondary antibody for 4 hours at room temperature. After last washes in 1× PBS, sections were mounted on glass slides using the mounting medium (Vector Laboratories) with or without 4′,6-diamidino-2-phenylindole (DAPI) and cured overnight in dark at room temperature. Antibodies and dilutions used are as follows: rabbit anti-GLuc (1:1,500, Nanolight Technology) and Alexa 488 secondary antibody (1:500, Life Technologies). All images were acquired by the BZ-X810 fluorescence microscope (Keyence).

### Statistical analysis

Two-tailed paired *t* test with unequal variance was used to compare two datasets when comparing means, or ratio-paired *t* test was used to compare fold changes in serum marker levels with FUS-BBBO marker recovery. One-way ANOVA or Kruskal-Wallis tests with Tukey post hoc test was used to compare means between more than two datasets in any other case, depending on whether the SDs of compared groups were significantly different in Brown-Forsythe test. Kruskal-Wallis was selected for c-Fos measurement data due to showing significantly nonnormal distribution (Shapiro-Wilk test for the CNO-on-target FUS group, *P* = 0.0006). All *P* values were determined using Prism (GraphPad Software), with the statistical significance represented as ns (not significant), *P < 0.05, **P < 0.01, ***P < 0.001, and ****P < 0.0001. The sample and effect sizes were calculated using G*power 3 software (*82*), assuming 80% power and α value of 0.05. In the absence of previous data that could be used for calculation of effect size, we relied on sample sizes used in invasive measurement of tissue gene expression (*83*) (for data in Figs. 2A and 3), or in noninvasive chemogenetic neuromodulation with gene delivery using FUS-BBBO (*30*) (for data in Fig. 5, E and F).

### Stereotaxic injection of AAV

AAV were injected into the brains using a microliter syringe equipped with a 34-gauge beveled needle (Hamilton) installed to a motorized pump (World Precision Instruments) using a stereotaxic frame (Kopf). For AAV, PHP.eB serotype was used. For chemogenetic neuromodulation experiments, three different AAVs were delivered. AAV encoding *hSyn-hM3Dq*-RAM-d2tTA and RAM-*GLuc*-IRES-*GFP* were delivered to the left hemisphere caudate putamen (CPu) in the striatum in the following ipsilateral coordinates: [anteroposterior (AP), +1.0 mm; mediolateral (ML), +2.0; and dorsoventral (DV), +3.8 mm], (AP, +0.5 mm; ML, +1.5,; and DV, +3.0 mm), and (AP, −0.5 mm; ML, +2.5; and DV, +3.5 mm). AAV encoding hSyn-CLuc was delivered to the right hemisphere CPu in the striatum in the contralateral coordinates. For each mouse, 1.0 × 10^9^ viral particles of each AAVs were delivered correspondingly. For AAV injections, 568 nl of AAV (*hSyn-hM3Dq*-RAM-d2tTA) and 116 nl of AAV (RAM-*GLuc*-IRES-*GFP*) were prepared in a cocktail and 684 nl total volume was injected each site. AAV (*hSyn-CLuc*) alone was injected 500 nl each site. The injection speed was 250 nl min^−1^. The needle was kept at the injection site for 10 min owing to the relatively high volume of injection.

### Dox control of gene expression

For chemogenetic neuromodulation experiments, mice were placed on Dox chow (40 mg kg^−1^) (Bio-Serv) 24 hours before intracranial injection of AAVs. Dox chow was maintained for 7 days after the AAV injection. Dox chow was removed 48 hours before inducing chemogenetic activation.

### Chemogenetic activation of neuronal activity

Water-soluble CNO (Hello Bio #HB6149) was dissolved in saline (Hospira) at 1 mg ml^−1^ and stored at −20°C until use. To induce chemogenetic activation of mice expressing hM3Dq, CNO was injected intraperitoneally at 5 μg/g mouse.

### Blood collection for luciferase assay

For the groups of mice in GLuc in vivo half-life measurement experiment, baselines were collected 5 min before GLuc intravenous injection. Experimental samples were collected 7.5 min postinjection in common and 30-, 60-, and 120-min postinjection based on their assigned groups. Up to 50 μl of blood was collected for each time point, and each mouse underwent a maximum of three blood collections, including the baseline measurement. For the groups of mice measuring transduction after systemic AAV delivery, baselines were collected before subcutaneous analgesic drug injection in the ultrasound insonation procedure. Experimental samples were collected 7.5 min after an ultrasound insonation session. For the group of mice in GLuc recovery in extended time frame experiments, along with baseline measurement, experimental samples were collected twice, 7.5 and 120 min after an ultrasound session, 50 μl in volume per collection. For the groups of mice where we used REMIS to measure chemogenetic activation, baselines were collected twice; before intracranial AAV injection and before CNO intraperitoneal administration. Experimental samples were collected 7.5 min after an ultrasound insonation session. Up to 100 ml of blood was collected for each sample, with the last sample always being a terminal collection, followed by immediate cardiac perfusion.

### Hematoxylin staining

Sectioned brain samples were stored in 1× PBS and prepared in water 10 min before staining. Samples were dehydrated in 100% ethanol and 95% ethanol, 1 min each. Then, the samples were rinsed in water for 1 min. Next, samples were immersed in 0.4× diluted Mayer’s modified hematoxylin solution (Abcam, #64795) for 1 min and rinsed again in water for 3 min. Nuclear stain was completed by immersing samples in the Blueing reagent(Abcam, #66152) for 20 s. Last, samples were rinsed in water for another 3 min. Samples were rehydrated in water for another 10 min before mounting.

### Cell positivity quantification

C-Fos images were taken on a Keyence BZ-x810 using the 20× objective lens. Each brain section had three images taken, one on top of the other in a vertical line inside of the blood brain barrier opening guided by the EBD signal (Fig. 5D). Images were counted manually in Zen Software (J.S.T.). Each image was loaded in and the upper limit of the histogram was reduced to 15,000 of 65,536 for every image to aid in visualization of all cells. Blinding was not possible due to the stark differences between the experimental group and controls.

GLuc cell counts images were taken on a Keyence BZ-X810 using the 20× objective lens. Single hemisphere images were taken and counted in Zen software by adding a horizontal only grid line spaced by (400 pixels) vertically between each line. Only green and blue cells that touched this line were counted. Green cells were taken in the 488 channel and designate GLuc expression. Blue cells were taken in the DAPI channel and were stained using DAPI containing mounting media. The experimenter (R.Z.W.) was blinded to identity of the groups.

Cell specificity for GLuc- and NeuN-positive images were taken on a Zeiss LSM-800 confocal microscope. NeuN was stained using Novus Biologicals RBFOX3/NeuN antibody conjugated to Alexa Fluor 405. GLuc was stained with anti-GLuc antibody (Nanolight Technology) and an Alexa Fluor 594 secondary antibody. GLuc and NeuN images were taken using the 20× objective lens on the confocal, and each image was overlaid and counted manually using Zen software. Total of 11 sections across four mice was stained and counted (two to three sections per mouse).
