# Supplementary material for: Acoustically targeted measurement of transgene expression in the brain
Source: Sci Adv. 2024 Aug 7;10(32):eadj7686. doi: 10.1126/sciadv.adj7686 (PMC11305388; doi:10.1126/sciadv.adj7686)
Supplement: Supplementary file 1 — Figs. S1 to S7 Supplementary source data tables [file sciadv.adj7686_sm.pdf]

Science Advances
AAAS

Supplementary Materials for

Acoustically targeted measurement of transgene expression in the brain

Joon Pyung Seo *et al.*

Corresponding author: Jerzy O. Szablowski, jszab@rice.edu

Sci. Adv. **10**, eadj7686 (2024)
DOI: 10.1126/sciadv.adj7686

This PDF file includes:

Figs. S1 to S7
Supplementary source data tables

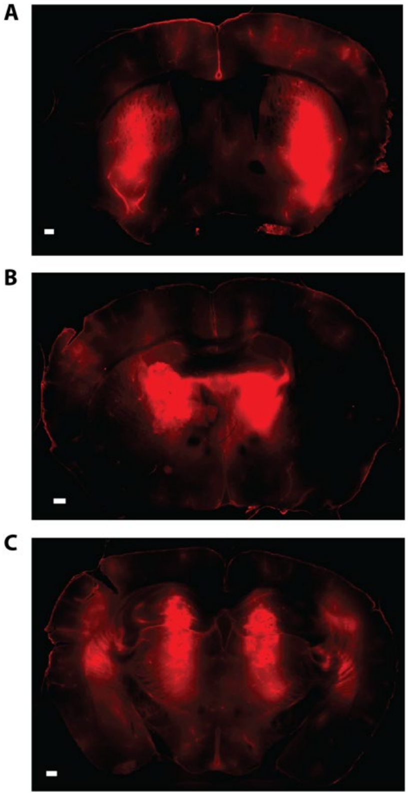

Supplementary Figure S1. BBB opening in high ultrasound pressure group AAV(+) FUS(+). Representative brain section on red channel visualizing EBD extravasation and approximating the targeted regions of the brain. The volume targeted was calculated from the full-width-half-maximum (FWHM) pressure profile, which was approximated as an an ovoid with the length of 5.4 mm and width of 0.9 mm, for a total volume of 2.3 mm^3. Assuming the C57BL6j mouse brain volume of 508.91 mm^3 (*87*), 8 sites at 2.3 mm^3 each would result in 3.6% of the targeted volume. Scale bars are 500 microns.

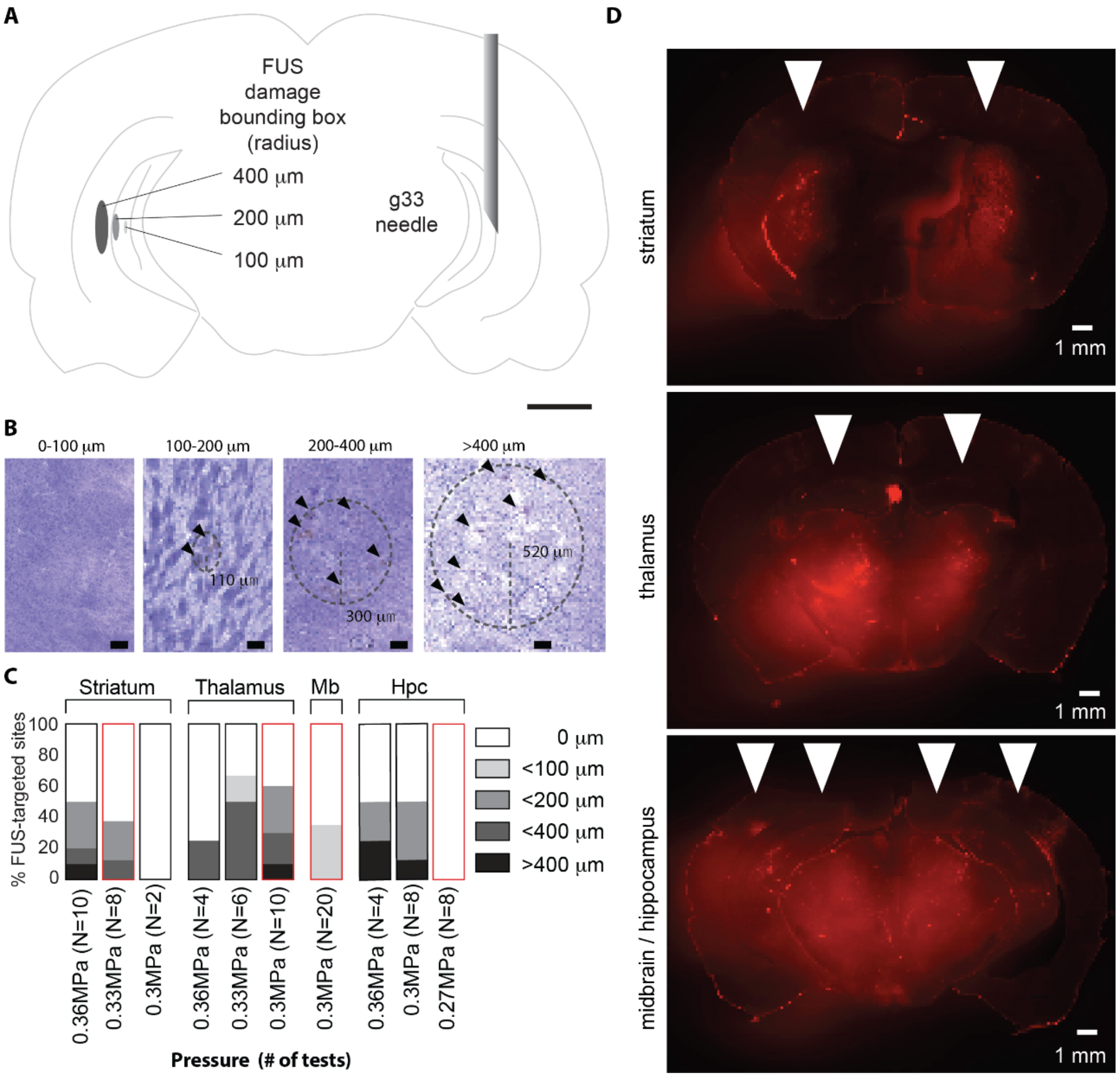

Supplementary Figure S2. Optimization of FUS peak negative pressure levels for different brain regions. (a) The size of areas within which any damage is contained are shown for illustration purposes and have the approximate shape of an ultrasound pressure field. Throughout this area, only some spots show damage. For comparison, a g33 needle is shown to visualize the tissue displacement needed for molecule delivery or biopsy. (b) Quantification of the maximum radius of the histological damage observed in mice. Histological damage was measured in 4 different areas of the brain; striatum, thalamus, midbrain (Mb), and hippocampus (Hpc). Optimal peak negative pressures were adjusted by steps of 0.03MPa down from 0.36MPa to find a set of conditions that resulted in no or minimal damage while maintaining BBB opening. Conditions shown in red boxes were chosen for all experiments, unless otherwise noted. **(d)** Representative images showing extent of the BBB opening (EBD extravasation, red, highlighted with arrowheads) with the chosen parameters. Scale bars are 1 mm.

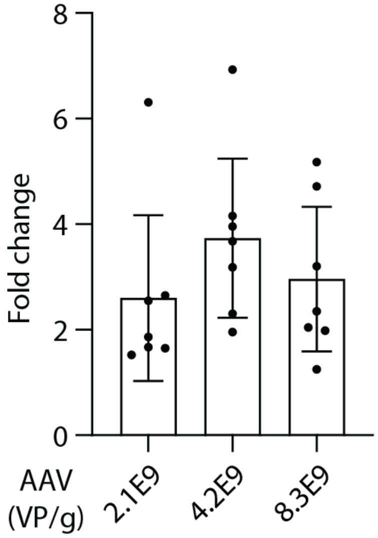

Supplementary Figure S3. Comparison of fold changes in serum concentration of GLuc induced by FUS-BBBO. AAV PHP.eB carrying GLuc under hSyn1 promoter was injected intravenously at different doses 2.1 x 10^9 viral particles (VP) per gram of body weight, or double (4.2x10^9 VP/g) or quadruple that dose (8.4x10^9 VP/g). After 3 weeks, FUS-BBBO was performed to release GLuc from the brain. The pressure used for release was optimized for safety, and included 0.33 MPa in the striatum, 0.3 MPa in the thalamus and midbrain, and 0.27 MPa in the hippocampus (P=0.4184; F=0.9148; one-way ANOVA).

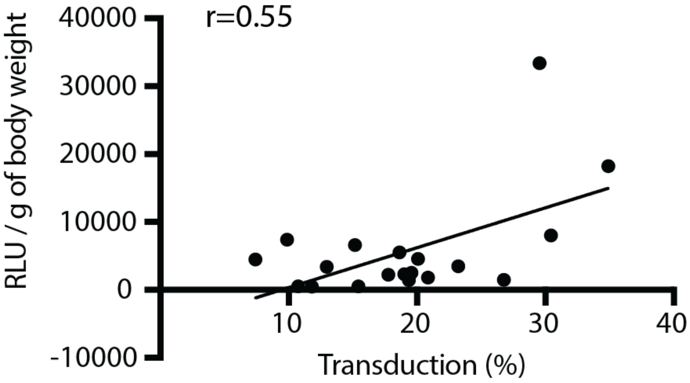

Best-fit values	
Y intercept	-5537
Slope	587.5
95% CI (profile likelihood)	
YIntercept	-14827 to 3754
Slope	132.9 to 1042

Supplementary Figure S4. Correlation between the post-FUS-BBBO GLuc luminescence levels in the serum and transduction. The GLuc luminescence levels were normalized to the body weight to account for differences in the circulating blood volume of mice. N=19 mice were tested.

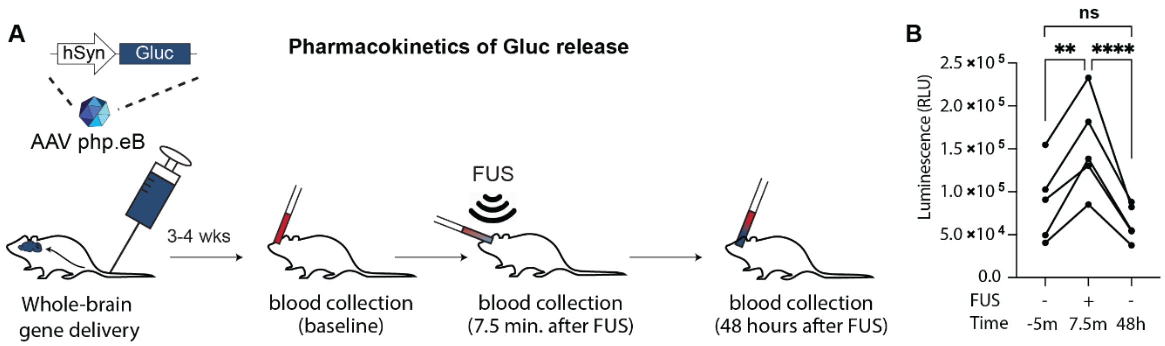

Supplementary Figure S5. REMIS signal is temporary and returns to baseline 48 hours after FUS-BBBO. Blood samples were collected from GLuc-AAV+ mice before, and after FUS-BBBO. After 48 hours, blood samples were collected again from each mouse, to evaluate levels of GLuc luminescence at each timepoint. We found a significant increase in the serum GLuc signal level after FUS-BBBO (1.9±0.5-fold, mean with 95% CI, p=0.0068, t=5.128, df=4, two-tailed ratio paired t-test), which was followed by a significant decrease in the GLuc signal 48 hours later (2.4±0.3-fold, mean with 95% CI, p<0.0001, t=16.51, df=4, two-tailed ratio-paired t-test). GLuc signals in the final blood samples were not statistically significantly different than the baseline (0.8±0.2-fold, mean with 95% CI, p=0.14, t=1.838, df=4, two-tailed ratio-paired t-test).

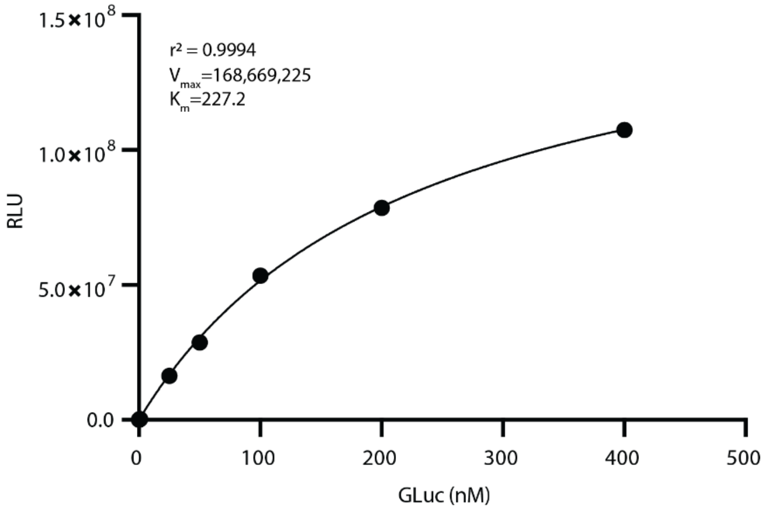

Supplementary Figure S6. Standard curve for the luciferase signal using our protocol, fitted to Michaelis-Menten model. For the assay, 15 µl of mouse serum was placed in a black 96-well plate. Bioluminescence of GLuc was measured by injecting the samples with 50 µl of 20 µM coelenterazine (CTZ) (Nanolight Technology) dissolved in luciferase assay buffer using an injector in a Tecan M200 microplate reader (Männedorf, Switzerland). (n=3 technical replicates tested)

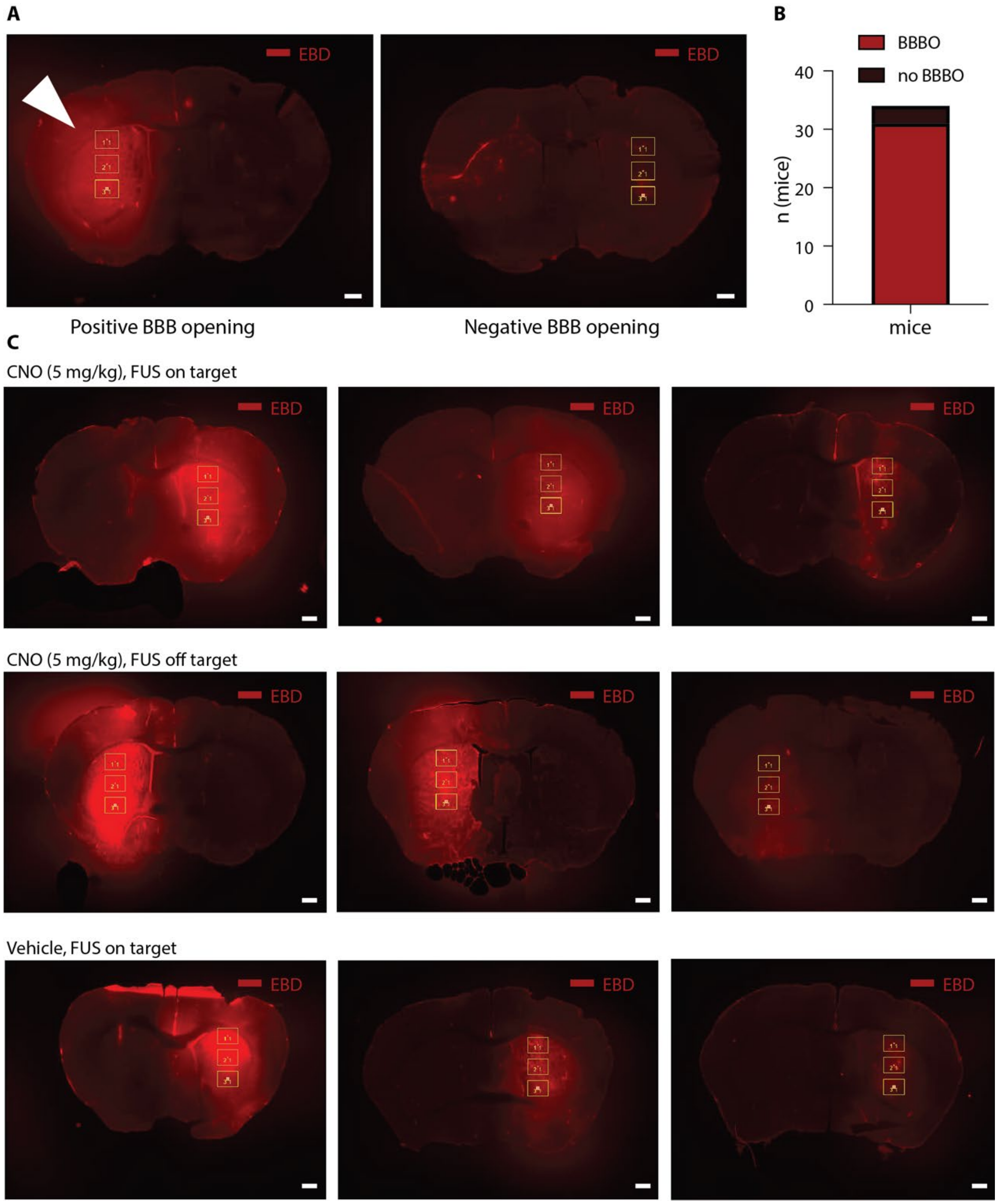

Supplementary Figure S7. Validation of the BBB opening during measurement of *c-Fos* activity with REMIS. (a) Within 20 minutes of the BBB opening, mice were administered Evans blue dye (EBD, red) I.V. which can cross through an opened BBB from the blood into the brain. EBD shows red fluorescence that can be assessed to evaluate the quality of BBB opening. The presence or lack of BBB opening was assessed qualitatively as positive or negative. Two representative images showing opening or its lack are shown. **(b)** Overall, over 91.2% (31 out 34 mice) showed successful BBB opening in the targeted sites. **(c)** Examples of positive BBB opening sites for each group showing the approximate range of possible results (N=3 per group). Scale bars are 1 mm for all panels.

Supplementary Source Data Tables

Figure 2b

	AAV- FUS-	AAV- FUS+	Fold change:
GLuc signal	51669	266227	5.152547949
	18351	81100	4.419377691
	14037	44432	3.165348721
	55842	409246	7.328641524
	76430	336404	4.401465393
	67032	170720	2.546843299
	136005	1326339	9.752134113
	45058	319694	7.09516623
	19969	229219	11.47874205

Figure 2c

	AAV- FUS-	AAV- FUS+	Fold change:
GLuc signal	2080	2181	1.048557692
	2228	2202	0.988330341
	2085	1954	0.937170264
	3246	4398	1.354898336
	2044	2408	1.178082192

Figure 2d

	AAV FUS High	AAV FUS Low
Fold change	5.15	4.72
	4.42	5.18
	3.17	2.05
	7.33	1.25
	4.40	1.98
	2.55	2.35
	9.75	3.20
	7.07	
	11.48	

Figure 2e

	FUS-	FUS+	Fold Change
2.1E9 VP/g	6935	12914	1.862148522
	8131	13608	1.673594884
	32157	52921	1.645707

	15578	23742	1.52407241
	32215	85335	2.64892131
	18474	116512	6.30680957
	15823	40370	2.551349302
		Average	2.601800428
	FUS-	FUS+	
4.2E9 VP/g	52197	206503	3.956223538
	8593	16789	1.953799604
	43248	137639	3.182551794
	19107	132400	6.929397603
	19753	82104	4.156533185
	17120	62931	3.675876168
	16062	36974	2.301954925
		Average	3.736619545
	FUS-	FUS+	
8.4E9 VP/g	184042	868273	4.717798111
	84473	437558	5.179856285
	19242	39376	2.046356928
	77661	97063	1.249829387
	18150	35951	1.98077135
	64745	152171	2.350312765
	35895	114685	3.195013233
		Average	2.959991151

Figure 2f

Low	Medium	High
20.19577	9.886057	30.42353
17.75567	19.36126	20.85665
9.487139	19.0066	23.21121
7.420432	20.07913	26.7627
12.96511	15.15752	34.90678
19.55628	18.63339	29.55151
15.43336	10.74591	
11.81946		

Figure 3a

Neun(+)	Neun(-)
186.6667	1.666667
172.3333	3
105.6667	3.666667
144.5	2

Figure 4b

	Control	7.5 min	120 min
Serum RLU	94529	219552	922567
	97586	274058	426807
	136836	366171	424003
	103978	421765	272534
	123733	217127	216070
	123695	266088	314642

Figure 4d

	7.5 min	30 min	60 min	120 min
Retention of GLuc	105.5616	22.0811	0.9883	0.2624
	62.2732	11.6022	0.4852	0.3583
	113.1649	12.5571	0.3341	0.0883
	254.4298	9.8915	0.4382	0.1729
	37.8801	2.8109	0.9399	0.5686
	111.9186			
	28.8665			
	42.8419			
	106.1271			
	168.2389			
	81.1630			
	103.0195			
	56.5171			
	48.2041			
	179.7935			

Figure 5e

	**CNO+ FUS+**	**CNO+ FUS-**	**CNO- FUS+**
Fold change over baseline	45	4.4782609	7.6538462
	28.21	7.95	10.333333
	7.34	1.7096774	1
	5.17	2.2857143	7.6551724
	93.18	10.358974	8.7857143
	10.86	4.3369565	2
	24.52	30.689655	20.431818
	310.68	13.686441	6.9921875
	82.98	16.410714	11.137931
	19.85	26.558608	7.8891753

105	7.6453901
78.75	
67.12	

Figure 5f

CNO ON Target	CNO FUS-	CNO- FUS+
151.6666667	0.555555556	15.33333333
80.66666667	3.444444444	11.22222222
88.88888889	3.666666667	2.777777778
70.11111111	0.111111111	17.66666667
62.11111111	1.222222222	16.33333333
143.4444444	3.333333333	19
101.4444444	0.888888889	14.66666667
143.2222222	2.555555556	19.55555556
125.1111111	3	9.444444444
212.4444444	0.444444444	11.33333333
118.3333333		19.33333333
75.33333333		

Supplementary Figure S1.

Targeted Area of the Brain	Thalamus	Striatum	Midbrain and Ventral Hippocampu s	Total
Number of FUS targeted sites on single coronal section	2	2	4	8
Number of successful BBBO sites from EBD histology image	2	2	4	8
Number of successful BBBO sites from EBD histology image	1	2	4	7
Number of successful BBBO sites from EBD histology image	2	2	3	7
Number of successful BBBO sites from EBD histology image	2	2	4	8
Number of successful BBBO sites from EBD histology image	1	2	4	7
Number of successful BBBO sites from EBD histology image	2	2	4	8
Number of successful BBBO sites from EBD histology image	2	1	3	6
Number of successful BBBO sites from EBD histology image	2	2	4	8
Number of successful BBBO sites from EBD histology image	2	2	3	7

Supplementary Figure S2c

Damage bounding box	0.36 Str	0.33 Str	0.3 Str	0.36 TH	0.33 TH	0.3 TH	0.3 MB	0.36 HPC	0.33 HPC	0.3 HPC
0	5	5	2	3	2	4	13	2	4	8
<100	0	0	0	0	1	0	7	0	0	0
<200	3	2	0	0	0	3	0	1	3	0
<400	1	1	0	1	3	2	0	0	0	0
>400	1	0	0	0	0	1	0	1	1	0

Supplementary Figure S3

2.1x10E9 AAV/g mouse (N=7)							
Low Pressure	1.86	1.67	1.65	1.52	2.65	6.31	2.55
4.1x10E9 AAV/g mouse (N=7)							
Low Pressure	3.956	1.954	3.183	6.93	4.157	3.676	2.302
8.3x10E9 AAV/g mouse (N=7)							
Low Pressure	4.718	5.18	2.046	1.25	1.981	2.35	3.2

Supplementary Figure S4

Weight (g)	Sex	% GLuc/DAPI (Average over ST/TH/MB)	Viral dose	RLU FUS(-)	RLU FUS(+)
18	Female	17.7556653	2.1E9 VP/g	15823	40370
26	Male	7.42043202	2.1E9 VP/g	18474	116512
25	Male	12.9651122	2.1E9 VP/g	32215	85335
21	Female	19.5562767	2.1E9 VP/g	32157	52921
27	Male	15.4333649	2.1E9 VP/g	8131	13608
26	Male	11.8194554	2.1E9 VP/g	6935	12914
19	Female	30.4235336	8.3E9 VP/g	64745	152171
20	Female	20.8566531	8.3E9 VP/g	18150	35951
28	Male	23.211207	8.3E9 VP/g	77661	97063
27	Male	26.7626976	8.3E9 VP/g	19242	39376
24	Male	34.9067792	8.3E9 VP/g	84473	437558
26	Male	29.5515066	8.3E9 VP/g	184042	868273
28	Male	9.886057	4.2E9 VP/g	52197	206503
26	Male	19.3612643	4.2E9 VP/g	16062	36974
27	Male	19.0065963	4.2E9 VP/g	17120	62931
18	Female	20.079126	4.2E9 VP/g	19753	82104
20	Female	15.1575168	4.2E9 VP/g	19107	132400
25	Female	18.6333941	4.2E9 VP/g	43248	137639
31	Male	10.7459137	4.2E9 VP/g	8593	16789

Supplementary Figure S5

FUS-	FUS+0h	FUS+48h
154601	232893	82153
49514	138478	54946
90642	130244	53977
102680	181582	88017
40430	85070	37599

The following animal was excluded from further analysis, due to technical limitations as described in the section entitled “Released marker recovery pharmacokinetics after insonation”.

FUS-	FUS+0h	FUS+48h
54530	26327	12333

Supplementary Figure S6

GLuc dose	GLuc activity
400	1.07E+08
200	78617554
100	53417403
50	28731949
25	16362738
1	310049.6
0.5	127175.4
0.25	79943.4

Supplementary Figure S7

	Opening present	Opening absent
CNO on target	12	1
CNO off target	8	2
Vehicle on target	11	0
